# Supplementary material for: Synthesis of New Star-Shaped Liquid Crystalline Cyclotriphosphazene Derivatives with Fire Retardancy Bearing Amide-Azo and Azo-Azo Linking Units
Source: Int J Mol Sci. 2020 Jun 16;21(12):4267. doi: 10.3390/ijms21124267 (PMC7352503; doi:10.3390/ijms21124267)
Supplement: Supplementary file 1 [file ijms-21-04267-s001.pdf]

Supplementary Materials – DSC thermogram of compounds **6a–e** and **8a–e**

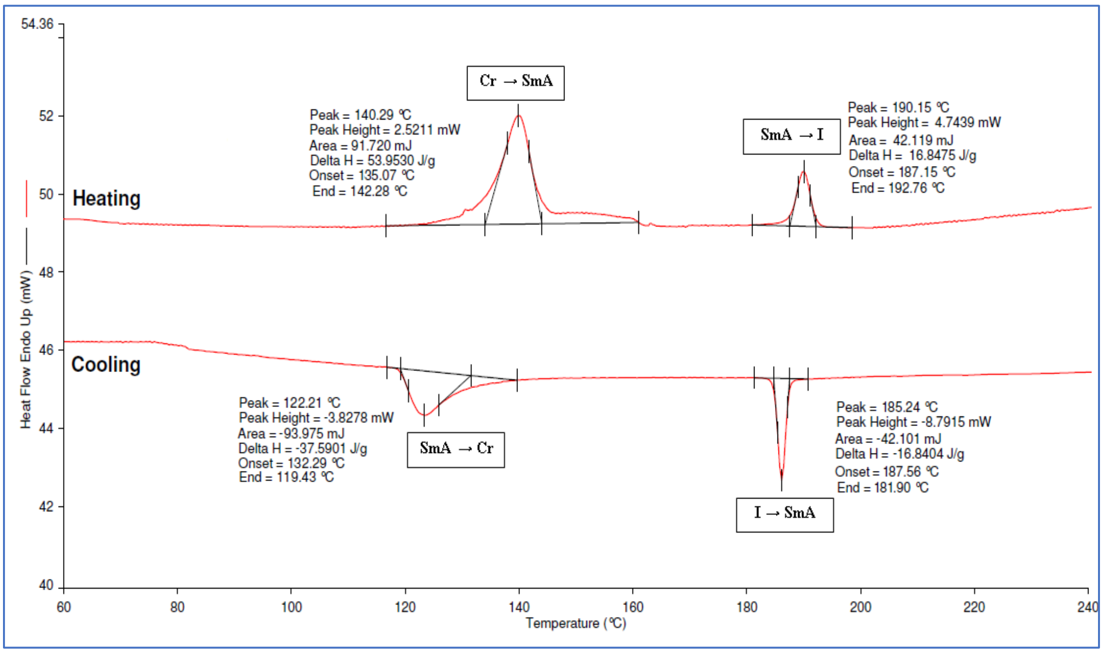

Figure S1. DSC thermogram of compound **6a**.

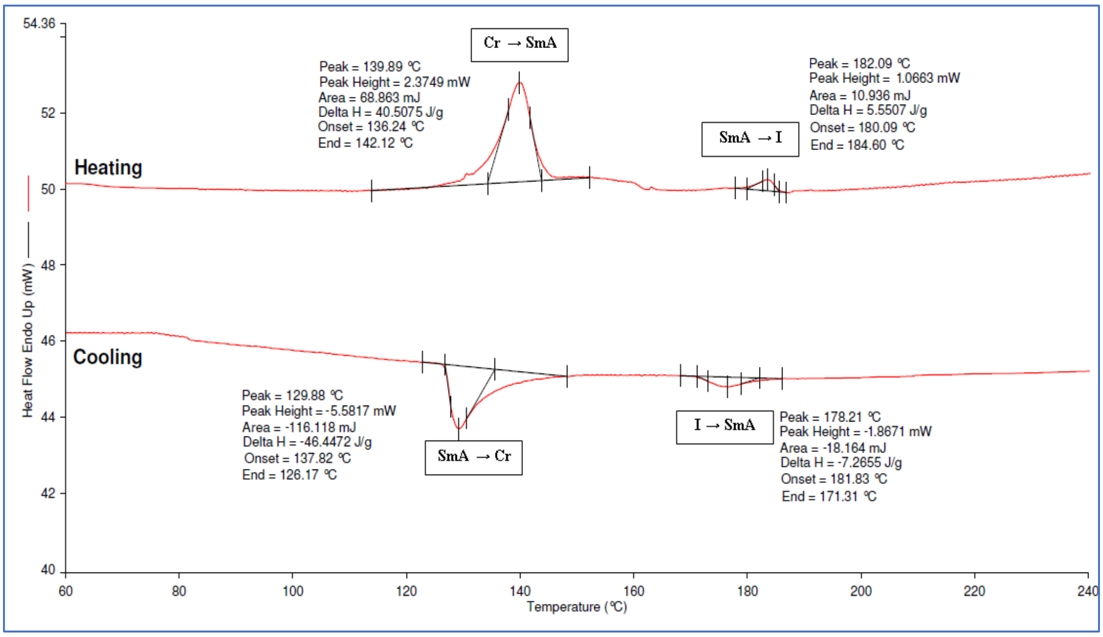

Figure S2. DSC thermogram of compound **6b**.

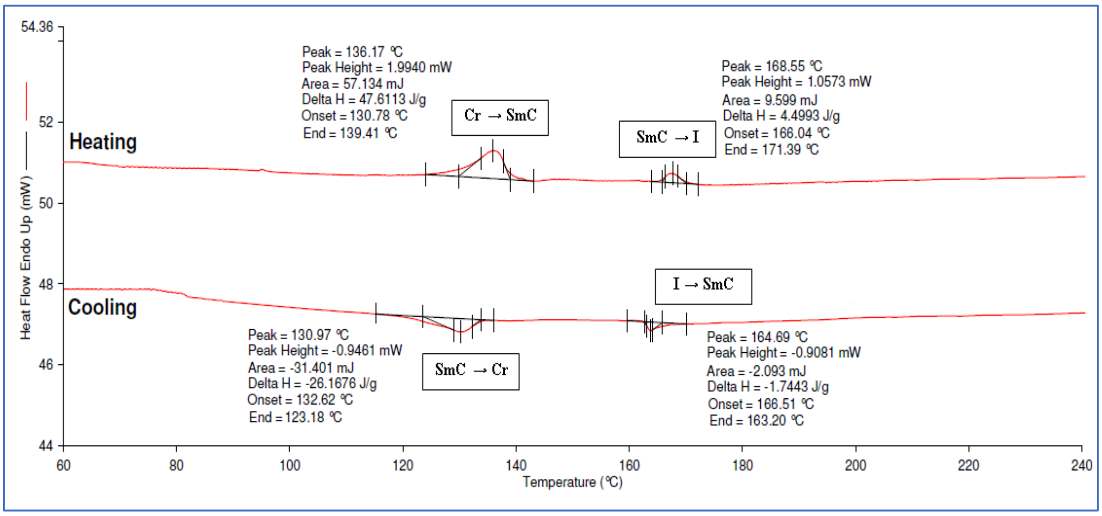

Figure S3. DSC thermogram of compound **6c**.

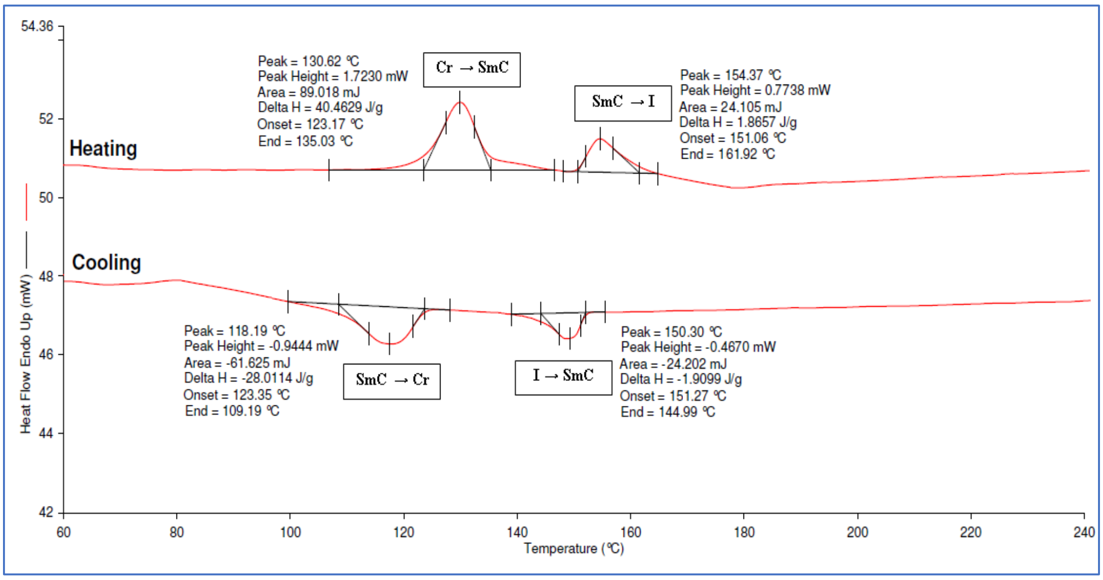

Figure S4. DSC thermogram of compound **6d**.

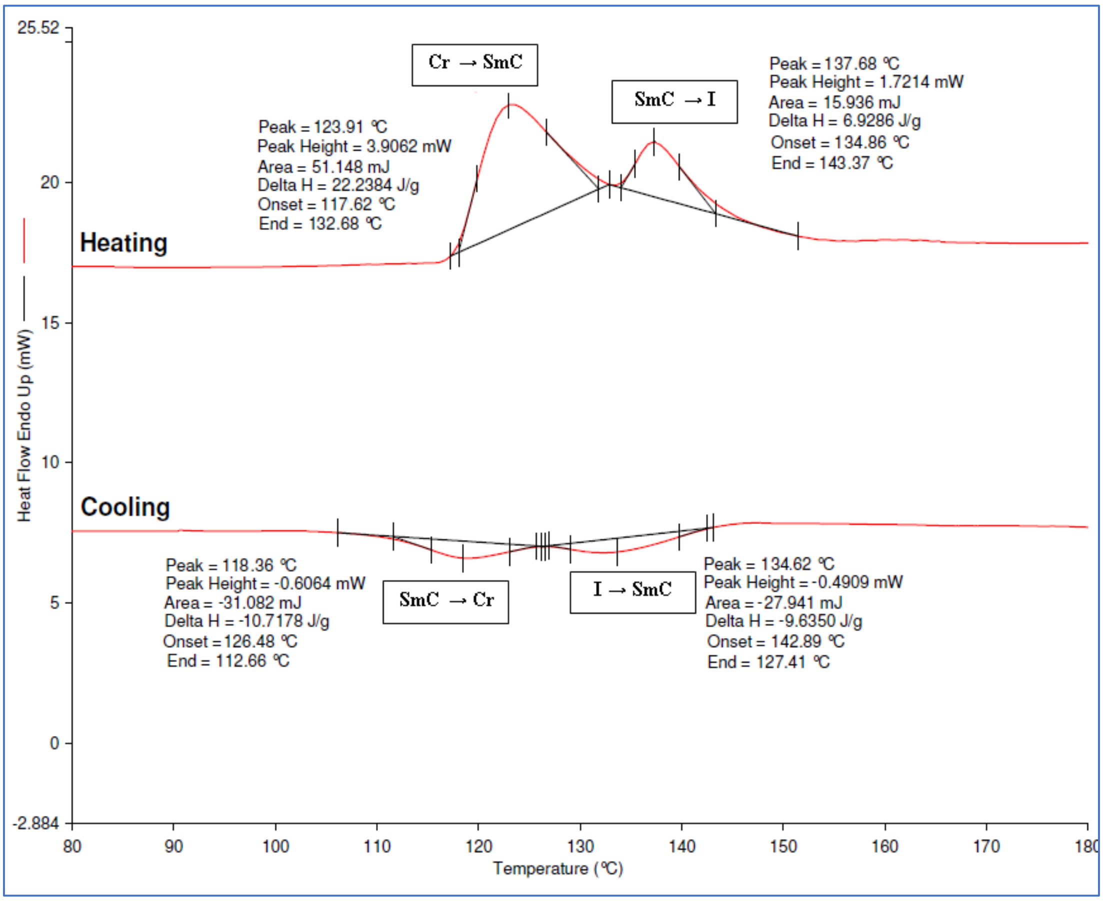

Figure S5. DSC thermogram of compound **6e**.

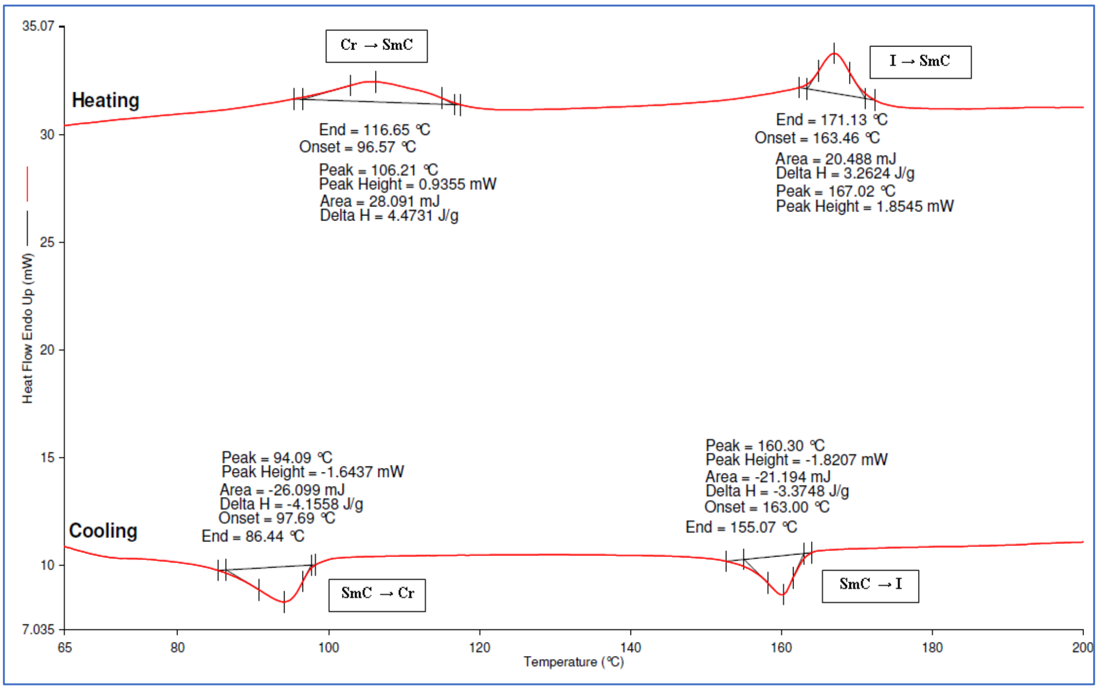

Figure S6. DSC thermogram of compound **8a**.

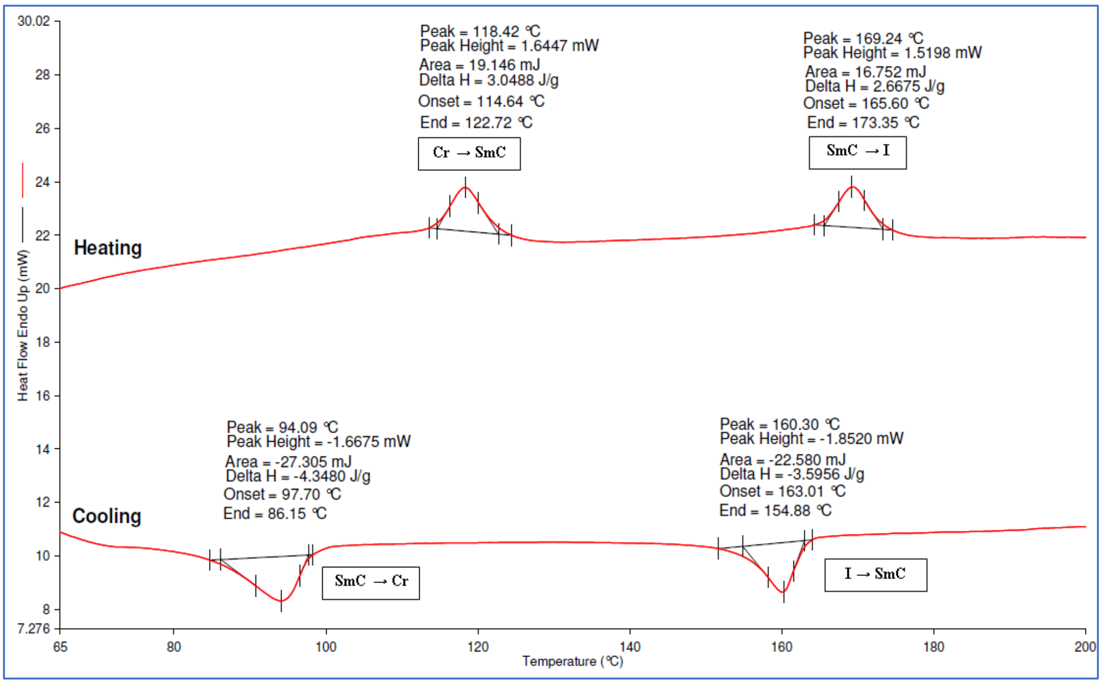

Figure S7. DSC thermogram of compound **8b**.

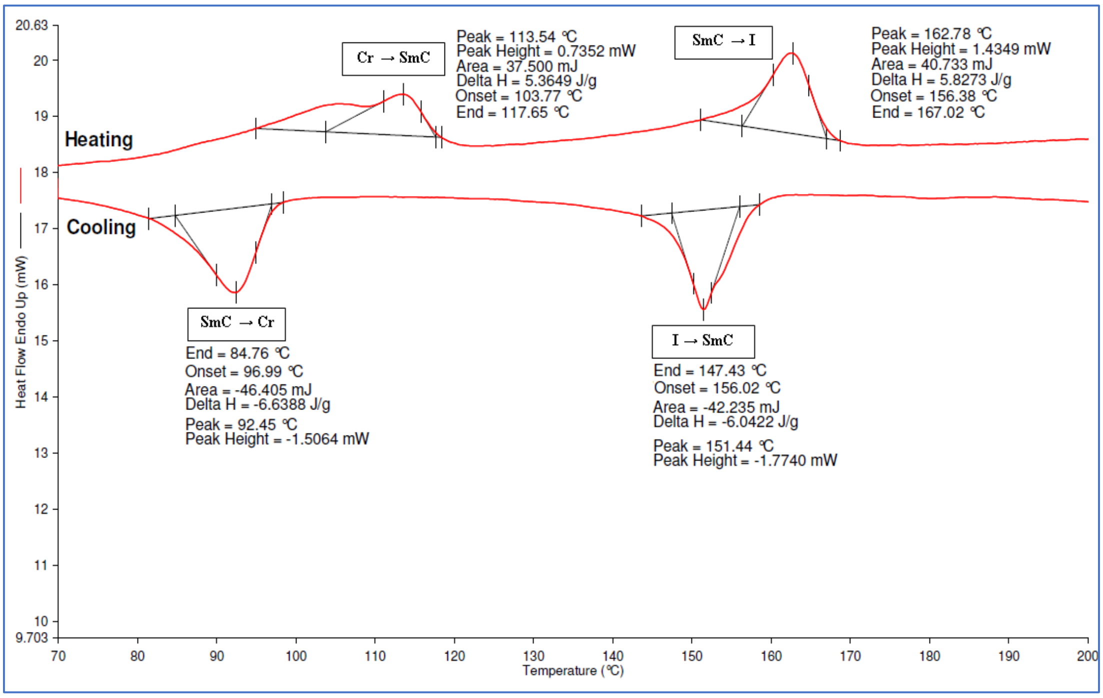

Figure S8. DSC thermogram of compound **8c**.

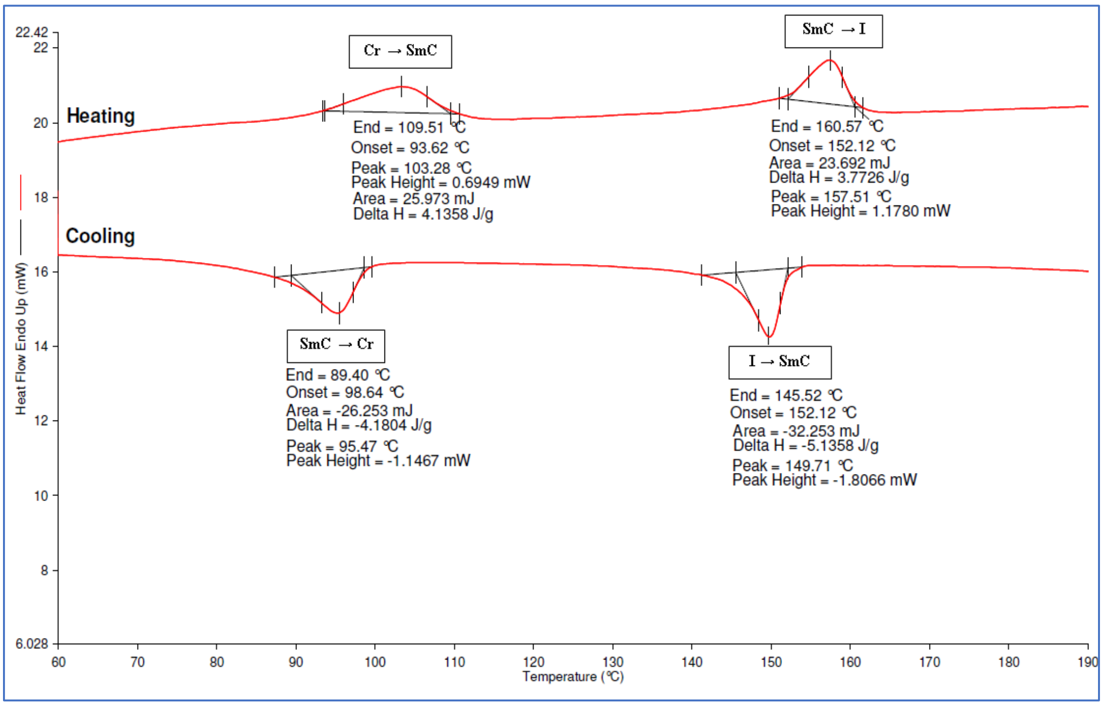

Figure S9. DSC thermogram of compound **8d**.

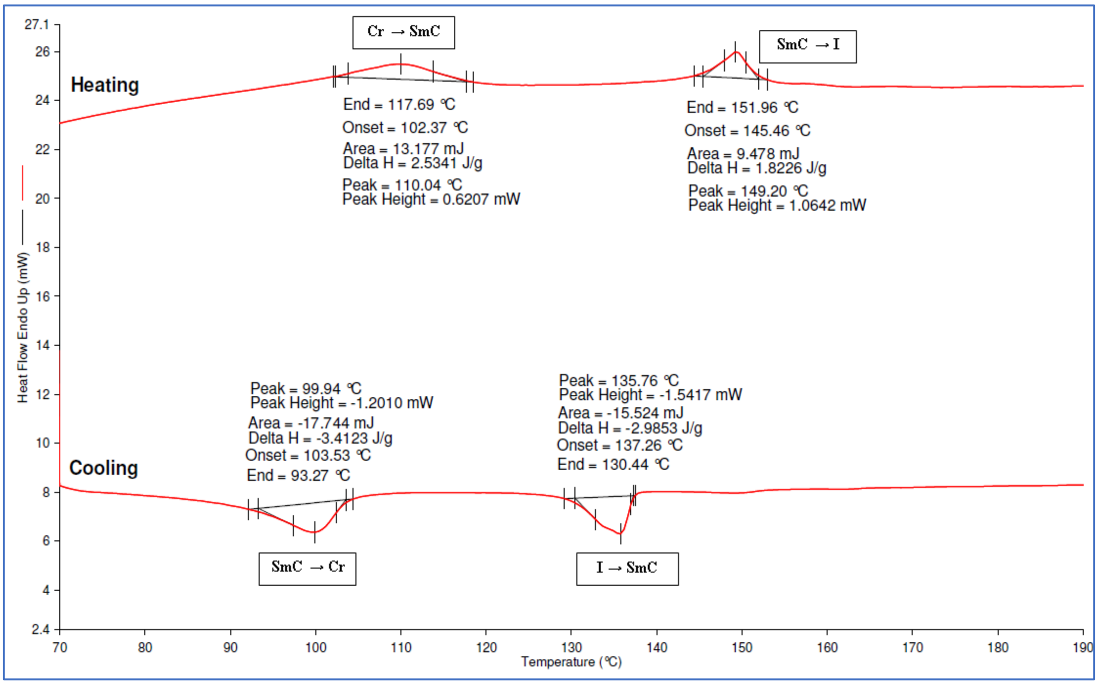

Figure S10. DSC thermogram of compound **8e**.
